# Significance of gain and loss of chromosomal abnormalities in AML: an Indian experience

**DOI:** 10.1186/1755-8166-7-S1-P45

**Published:** 2014-01-21

**Authors:** Pina J Trivedi, Manisha M Brahmbhatt, Dharmesh M Patel, Esha N Dalal, Geeta Joshi, Prabhudas S Patel

**Affiliations:** 1 Cell Biology Division, Gujarat Cancer Research Institute, Ahmedabad, India; 2 Department of Cancer Biology, Deputy Director, The Gujarat Cancer & Research Institute, Ahmedabad, Gujarat, India

**Keywords:** AML, Chromosomes, Karyotype, FISH

## Background

Acute myeloid leukemia (AML) is a heterogeneous group of disorder. Recurrent translocations are generally recognized to be a major parameter for prognostication in AML. Recurrent chromosomal translocation t(15;17),t(8;21) and inv(16) have good prognosis, whereas, loss and gain of different chromosomes and/or chromosome segments play a vital role by different mechanism in leukemogenesis. Cytogenetics is one of the most powerful independent prognostic indicators in AML. It serves to identify biologically distinct subsets of disease and has been widely adopted to provide the framework for risk-adapted treatment approaches. The aim of the present study was to appraise the clinical significance of numerical and structural chromosomal abnormalities in AML patients in terms of loss and gain of chromosomal material.

## Materials & methods

Bone marrow and peripheral blood lymphocytes of 321 AML patients were carried out by cytogenetics and FISH studies. Short term cultures and GTG banding were done for karyotyping. FISH and Multicolour FISH; were carried out as and when required as per standard protocols.

## Results

Out of all 321 patients, trisomy 8 showed the highest prevalence (n=14) and found as sole, complex and secondary change. Apart from most commonly observed recurrent chromosomal abnormalities, there were loss and gain of different chromosomes also observed. The loss of sex chromosome was observed in the highest frequency (n=20). Gain of whole chromosomes were; 8 (X23), 10 (X5), 19 (X7), 21 (X10), 22 (X6) and loss of X (X6), Y (X17). Mainly involved breakpoints in structural abnormalities were gain or loss of different chromosomes i.e. 1q (X8), 5q (X5), 8q (X4), 9q (X5), 11p (X5), 11q (X8), 17q (X9), and 22q (X 6).

## Conclusions

Study revealed that the, loss of chromosomal material was observed much more often than gain in AML with aberrant karyotypes. Hence, loss of tumor-suppressor genes may occur which may involved in mechanism of leukemogenesis. Numerical abnormalities in karyotype may affect gene-dosage and may play a significant role in the pathogenesis of AML. This study also highlights the importance of diagnostic cytogenetics as an independent prognostic factor in AML, providing the allocation for a stratified treatment approach of the disease.

